# Regression in the service of bibliotherapy—What can “Captain Underpants” teach us?

**DOI:** 10.3389/fpsyg.2024.1379115

**Published:** 2024-08-30

**Authors:** Sarit Ifrah

**Affiliations:** The Program for Hermeneutics and Cultural Studies, Interdisciplinary Studies Unit, Bar-Ilan University, Ramat Gan, Israel

**Keywords:** child psychotherapy, children's writing, reading, psychoanalysis, children's literature, potential space

## Abstract

Regression in the service of the ego is a unique phenomenon that often occurs within therapeutic settings. In the current study^1^, I show how it emerges within child therapy and how bibliotherapy manages to give it presence and thus to process it. The methodology that guided this study was based on a critical reading of psychoanalysis and bibliotherapy theories. In addition, the methodology is based on a therapeutic vignette aimed at demonstrating the qualities of bibliotherapy with children. I claim that bibliotherapy, based as it is primarily on the use of reading and writing processes, offers additional ways of processing and thinking about this phenomenon. The study provides an innovative contribution that is related to the interdisciplinary approach to therapy. There are important links between the two major disciplines examined in this study, psychoanalysis and bibliotherapy. Their intertwining generates interrelations and mutual inspiration. Moreover, this study adds to the theoretical and practical foundation of bibliotherapy and further establishes the understanding regarding the power of reading and writing processes to “relate the soul” within the analytical process.

## Creative processes through a psychoanalytical lens

### Primary and secondary processes

The psychoanalytical theory on creative processes begins with two main ideas: primary processes and secondary processes. Primary processes contain unconscious contents of thinking and imagination guided by the pleasure principle and considered primitive. This is a mental energy that flows freely and unimpeded from one representation to another (Freud, 1967; Laplanche and Pontalis, 2011). Secondary processes, in contrast, contain contents from a system that is close to consciousness as well as to the conscious system, which include rational and objective thinking controlled by logic. In this case, mental energy is invested in a stable way and only then does it flow under control. This facilitates mental experiences, where a range of routes for attaining satisfaction are examined (Freud, 2007; Eigen, 1983; Suler, 1980). The thinking of Freud (2007), Kris (1952), Rank (1990), and Ehrenzweig (1962) about artists and creative processes utilizes these concepts and further characterizes theories of creative and expressive therapy.

It is currently understood that the intertwining of secondary processes within primary processes is essential and that the two are not dichotomous (Milner, 2006; Noy, 1999). Marion Milner contends that the primary processes have changed their meaning and are currently perceived as part of the integrative functioning of the “self”; namely, they complement the secondary processes and are vital for them. She contends that the primary process is that which contains paradoxes and contradictions rejected by the secondary process because it itself is bound by logical thinking (Milner, 2006). Continuing Milner, Noy (1999) indicates that any thinking, perception, internal representation, or communication related to artistic activity is always enriched by various components of the primary process. These components usually enter current thinking through the unconscious systems, although many of the primary components are often conscious.

### Regression in the service of the ego

Ernst Kris was a psychoanalyst and art historian who investigated creative processes among artists. Observing artists, Kris (1952) understood that their work contains another aspect, a special dimension whereby they resemble children. He referred to Freud's structural model that includes the ego, the id, and the superego, wherein each part is responsible for other elements. The superego is the part responsible for judgment, ethics, and morality; it is like a supreme court that examines what is morally right. The id is the liberated, unrestricted, and unrestrained part that is entrenched in the principle of pleasure. Moreover, between them both is the ego, the mediating part, which is responsible for reconciling between the two [Freud, cited by Mitchell and Black (2006)]. Anna Freud investigated the defense mechanisms of the ego, when she described both their manner of action and their location along the developmental continuity. She emphasized the importance of the ego as an object of psychoanalytic inquiry worthy of separate research and indicated the spreading of ego processes to all areas of the personality's functioning [Anna Freud, cited in Mitchell and Black (2006)].

In the artistic context, the id is controlled by primary processes that represent contents from the unconscious, such as dreaming and imagining, namely, the artist who operates from the parts that belong to the primary processes is connected to flowing unsupervised mental energy. However, creative processes, as stated, also draw from secondary processes (Laplanche and Pontalis, 2011; Noy, 1999; Freud, 1967, 2007; Eigen, 1983; Suler, 1980). In the dialogue between the primary and secondary processes and in the context of their appearance in the psychoanalytical theory, Kris (1952) suggests that the organizing and supervising capabilities of the ego, and particularly its ability to control primary processes, are very important. What is the significance of this ability? What is its relationship with creative processes and how is it associated with therapy?

According to Kris, as an essential part of the creative process the artist retreats, mentally, to areas of existence that are childlike, uncontrollable, and playful. In this way he allows himself to be in contact with the most primitive, ancient, and uncommunicative urges, the urges of the id. These urges, as stated, rely on the principle of pleasure and are operated by it. This pausing of the artist in areas of the id's urges provides him with release, free unrestricted energy that allows him to investigate his inner world with its raw and irrational parts. This pausing affords the artist a wallowing in the primary materials of the psyche, but with a communicative intention—a constant arrow indicates the way out, after processing these raw materials and transforming them into a work of art. The pleasure the artist derives from the creative process is encompassed both by releasing the urges and by controlling them (Geldman, 1998; Noy, 2005). Kris coined the term “regression in the service of the ego” to accentuate the association between the ego's monitoring abilities, which the artist needs to create communicative art, and the urges of the id that pull him back and are required to investigate primitive psychic areas. According to Kris, the insane artist does not control his regression, rather he is overwhelmed by it, and therefore, his artistic products can be understood only through interpretation. His art serves a restitutive function more than a communicative function [Kris cited by Knafo (2002)].

Balint (2006), who was familiar with the work of Kris, found that regression in the service of the ego resembles regression aimed at recognition, where the patient regresses to primary behaviors. He posits that, given a holding environment that manages to hold the patient and withstand the destruction he causes, there will be regression. Such an environment is recognizing, patient, and tolerant, and it retains the conditions for the regression that occurs in the patient's inner world. He claims that Kris was interested in art and in regression focused on artistic creativity and did not direct his research at non-artists. According to Knafo (2002), who relies on Balint's regression aimed at recognition alongside Kris' artistic regression, it is possible to compare the therapeutic setting to art. Thus, when Kris wrote about regression he meant an intra-psychic act whereby the artist's regression is possible due to his or her attitude to craft. The structure of art, similar to the structure of the therapeutic setting, assumes the existence of boundaries within which the artist acts and creates. This structure serves as a holding, containing, and validating environment that allows the artist's psyche to operate within it relatively safely. This occurs similarly within psychoanalysis: The analyst interprets regressive patients in a certain way to help them resist being overwhelmed by regressive forces. This can be done only when the therapeutic environment is facilitative and containing, where the patient feels safe and protected. Similar to the analyst's interpretation, the artist too passes from a state of inspiration (regression) to a state of elaboration (which encompasses editing and criticism) and thus achieves a high level of organization and clarity (Knafo, 2002).

Shitrit-Gross (2012), an art therapist, related to the process of regression in the service of the ego in therapy with children. She describes a movement between working with dry materials such as plasticine and dough, which reflect control, order, and organization, to working with wet materials such as gouache and watercolors, which reflect flow and release. She claims that this movement is paramount to regression in the service of the ego, and in art therapy, this is done by working with materials (Shitrit-Gross, 2012). Dudek and Verreault (1989) explored how impulsive energy can be transformed into a creative product. In their view, children found to be creative, as evident in different tests of creativity, will be able to make extensive use of the process of regression in the service of the ego, as evident in how they relate to primary and secondary processes.

Segal, a British psychoanalyst and a student of Klein, also wrote about creativity. She understood that symbolic representation promotes the development of the ego, and she linked this to Klein's two positions, the depressive and the schizoid [Klein cited by Segal (2001)]. As she saw it, the ego develops and is transformed when depressive feelings surpass schizoid feelings, i.e., when the loss, separation from the object, ambivalence, and guilt are experienced painfully but still manage to remain tolerable. In this way, symbolism regulates, influences, and monitors the ability to communicate (Segal, 1957).

### The potential space—Winnicott

It is almost impossible to write about processes of artwork and creativity without referring to Winnicott's philosophy. Indeed, the term “potential space” establishes a wide frame of reference that helps understand the essence of creativity. This term, which has become a cornerstone of expressive and creative therapy, describes an area that contains phenomena and objects from the individual's inner world, besides phenomena from the external realistic world (Winnicott, 2009). Winnicott begins with the infant's transitional object, the object that the infant believes he himself found and that is charged with the emotional qualities of the mother, which provides consolation and respite in moments of distress. A continuation of this transitional object, as Winnicott (2009) sees it, is the cultural experience that includes play, creativity, literature, and art.

The transitional object is both a concrete and a symbolic object, a physical object that can be touched and realized but also an object that represents the quality of the mother. Through the transitional object, the baby begins to discern between fantasy and fact and between internal and external objects. As an extension of the child's self, the object embodies a process of forming the ability to accept difference and similarity, within the gradually consolidating symbolism. In this way, it helps the baby in the journey from the purely subjective, i.e., the idiosyncratic world, to the objective, i.e., the external world. In this way, the baby proceeds toward the experience (Winnicott, 2009). The potential space, which contains transitional phenomena and objects, is the protected area in which the creative self can operate and play: This is the area of experience from which art and culture derive. One who lives only in an inner world with no connection to external reality is immersed in oneself, while one who lives only in the objective reality indeed adapts to the world but does so at the cost of losing originality and passion. The vagueness of the intermediate area is that which implants experience in deep spontaneous sources within the self while also connecting it to the subjectivity of others [Winnicott, cited by Mitchell and Black (2006)]. Namely, when the baby plays with transitional objects, this constitutes the foundation for the cultural experience in adulthood.

### The transformational object—Bollas

Bollas (2018), who was influenced by the world of literature, understood the child's creativity through the connection with his mother. The infant experiences the mother as a transformational object in a stage when she is not yet identified as another. In maturity, he will continue to seek an object—aesthetic, human, creative—that signifies transformation, through which he will undergo transformation. In this way, the self is shaped and transformed by the environment, and a transition from the individual's isolation to human society is facilitated. In time, the mother's aesthetic is transferred to language, and at this stage, the infant's existence can be manifested.

“…As it was mother's style of transforming the infant's being that constituted the first human aesthetic, so too… **wording will handle and transform the moods of the self** and constitute further terms of that individual's personal aesthetic…”

(Bollas, 2018, p. 18, my emphasis).

Namely, the child undergoes a transformation through verbal language, which includes literary works besides creative writing. How can the transformation undergone by the individual be perceived? This is an intra-personal process, and only the individual can attest to having undergone a transformation. In my view, in children the transformation can be seen in their mental growth spurt that is related to many areas, including those of exposure to art. Thus, it is possible to speak to a child who is accompanied by a literary character; he speaks about the character and has experiences through it, and the character grants him confidence and capabilities. The transformation of the individual through art contains a strengthening of ego forces, capabilities, and trust in oneself (Bollas, 2018).

According to Bollas, the object that effects a change in the inner and external world, i.e., the transformational object, is identified as a process. Before serving as a subject for the baby, the mother is a source of change. As the baby develops, it understands that the mother is a separate subject and does not exist only to provide for its needs. However, the experience of the self transformed by the other remains a memory that can be realized again in aesthetic experience. Hence, in the aesthetic moment, when there is a deep connection between the subject and the object, culture constitutes a potential for realizing experiences of transformation (Bollas, 2018).

## Methodology

The methodology that guided this study is based on critical reading of bibliotherapy theories as well as on a therapeutic vignette.

### Ethics in research and in publication

To maintain ethics in research and in publication, I chose the format of a composite-type case description, where several patients and several vignettes are combined to form a single illustrative case. In this way, patients' identity was obscured and their confidentiality was maintained (Witztum and Margolin, 2002). In my opinion, this process sustains the rules of ethics for research and publication by therapists in general and by expressive and creative therapists in particular.

## Bibliotherapy—Literature review

The connection between psychoanalysis and literature presumes that the materials underlying both are similar to a large degree and that psychological and literary affiliations are compatible, as literary stories share a universal cross-cultural and cross-geographical core (Geldman, 1998; Lev Ari, 2011; Brooks, 1987). We assume that literature and psychological processes must be compatible; that aesthetic constructs that encompass literary images are congruent, in some way, with the psychological constructs they arouse and attract.

Bibliotherapy began from the tradition where librarians provided clients with reading recommendations according to the difficulties they were experiencing (Shechtman, 2010; Monro and Rubin, 1975; Shrodes, 1955) in the intuitive understanding that literature has the power to relieve patients' suffering through reading, provide significant insights, and generate change by reading and by discussing reading materials (Granot, 2020; Zoran, 2001; Koboby, 2008; Cohen, 1989; Monro and Rubin, 1975; Shrodes, 1955). Bibliotherapy as a therapeutic field based on psychodynamic theories, however, was initiated with the dissertation of Caroline Shrodes. Shrodes (1955), a psychologist and a professor of literature, claimed that in bibliotherapy there is an interaction between the reader's personality and the literature as a psychological field that encourages growth and adaptation. This claim was expanded in recent decades, and at present, bibliotherapy is recognized as one of the therapeutic approaches within expressive and creative therapy. As such, it is placed at the center of the therapeutic relationship processes of reading and writing in a literary text, mediated by a certified bibliotherapist (Zoran, 2001).

Many studies have indicated the centrality of bibliotherapy. These include working with a range of difficulties and disabilities, from working with survivors of sexual abuse and youth at risk, through working with loss, bereavement, and chronic illnesses, to working with the mentally ill (Kreuter and Reiter, 2014; Wright and Thiara, 2019; Yaniv et al., 2022; Mazza, 2017; Currier and Zimmerman, 2019; Daboui et al., 2018). These studies indicated a trend of gradually increasing use of reading and writing processes around the world.

### It's a bird! It's a plane! No, it's Captain Underpants

I meet with David once a week for therapy provided at school. He is in a regular fourth-grade classroom, and he has a learning deficiency that makes it hard for him to acquire reading and writing. We first met approximately 6 months after the waves of COVID-19 that restricted school attendance, where the previous 2 years had aggravated his learning disability and affected his emotional and social state not insignificantly.

Several months after beginning therapy, David started saying the words “pee” and “crap” and giggling. This surprised me because it was not appropriate for his age and developmental stage and was out of context. I wondered why these words were appearing now of all times. In one of our sessions, I remarked that the words reminded me of a book called *Captain Underpants*, with which David was familiar. When I asked whether he would like me to read aloud from the book he replied in the affirmative.

*Captain Underpants* is a comic book written by author and illustrator Pilkey (1999). It is a popular series with two main characters, Harold and George. The two try to survive elementary school through naughty acts and teasing. They write a comic story with a superhero who wears underpants, and they call him Captain Underpants^2^. Later, the two hypnotize the hated school principal, Mr. Krupp, and get him to think that he is Captain Underpants.

I read aloud several sections from the eighth part of *Captain Underpants and the Talking Toilets*. I was not surprised by his reading response, which included bursts of laughter; it was clear that he was enjoying it immensely. Every time the words “crap”, “pee”, “fart”, and “shit” were mentioned, and the book abounds with them, it produced waves of laughter.

When he calmed down, I suggested that he write a story inspired by the book. David agreed. We stapled several pages together to form a book-like object; he dictated the plot to me.

His story was as follows:

This is the story of David,

a boy who had many toilets,

stinky shit and people who sit in the lavatory.

Engaging with these contents aroused, as stated, much excitement and pleasure. The next sessions began by reading the story he had written, and for a certain time, David continued to give presence in his language, both in his written text and in speech, to his occupation with bodily excretions. During these sessions, he checked my responses, sometimes apologized for his words, and clearly felt ashamed. In this way, the regressive parts were given room and presence in the form of the literary work and subsequently in the story he had written. This wallowing in the infantile parts and in the early stages of development reflects the phenomenon of regression in the service of the ego (Kris, 1952). This involves regressing to an infantile past to touch its materials and engage in them only to let the self leave it reinforced and cohesive. Intersubjectively, from a state of assault against the relationship, the discourse on the bodily excretions became part of the relations between us, allowing attention to frightening and overwhelming parts of his psyche.

### Children's writing

In psychotherapy of the child, writing is a familiar practice (Gardner, 1970; Healey, 2019; Sunderland, 2017) and research on this topic is expanding. Freud (1977), the founder of child psychoanalysis, utilized various techniques, including imagination games, drawings, and writing poems and stories. Heller (1990), who was one of her patients, published the book *A Child Analysis with Anna Freud*, which presents her clinical journal, as well as the stories and poems he wrote while in therapy with her. Freud analyzed the stories, as she did the dreams presented by Heller (1990), as well as through psychoanalytical interpretation. Children's writing provides insights into their fields of interest and themes and serves as a means of processing these contents. It reflects a personal language, mental state, conflicts, and ways of coping (Sela and Raz, 2019; Segev-Shoham, 1998; Sunderland, 2017).

Roland Barthes contrasted the medium of speech with the medium of writing, where both represent mediums of communication. As he sees it, however, writing is a concrete action with aesthetic, linguistic, and social values (Barthes, 2004). Writing needs context and connections; in this way, the associativity and dynamism underlying the medium of speech are validated within the context of the story. The medium of writing, unlike the medium of speech, leaves behind something physical, a finished product, which can be revisited and observed.

Later, he created a comic strip and illustrated the characters in the story. He asked to write the back cover of the book, bearing the abstract:

*The story called: Captain Underpants and the Doctor*.

*The doctor was very angry at Captain Underpants. Do you know why? Because Captain Underpants confused him, annoyed him, and tried to hypnotize him*.

He illustrated the back cover of the story with markers, another physical medium, to supplement the written text. The medium of drawing, a domain he had mastered and which he used with no mediation, was his responsibility. The actual writing, a skill that he had not yet acquired, was mine. At the same time, David started writing single words on the stories; he slowly copied the name “Captain Underpants” from the cover of the original book. He wrote next to the characters he drew “fatspider” (fat spider), and “dinosormose” (dinosaur mouse) with spelling mistakes, standing up to his learning deficiency and giving it presence on the page. The medium of writing was embodied by the urge to know (epistemophilia) and to be understood in a world where membership requires proficiency in written language. In this way, he transformed his writing, appropriated it, but also shared it with me.

#### Text as a transitional object and as a holding environment

Berman (2003) contends that the space formed between the reader and the text is a “transitional space” such as that intended by Winnicott, and it is affected both by the work that is read and by the nature of the reader as an interpreting subject. The literary text is a type of transitional object and the act of reading it creates the transitional space within which the act of interpretation occurs. Zoran (2009), continuing Berman, emphasized that any bibliotherapeutic process, and primarily reading a literary text, reflects being in a transitional space. The individual experiences the reality of the book with all its descriptions and characters and these meet him (Zoran, 2009).

In other words, Berman and Zoran contend that the bibliotherapeutic process that includes reading and writing occurs in the transitional space and creates it. In this encounter, the individual undergoes a change, or in the words of Bollas (2018), a transformation. The literary text or the text written by the individual is an aesthetic object that leads to transformation.

Another concept received from Winnicott to understand the association between the text and the reader is the “holding environment”. Its essence is the initial relationship between the mother and her infant, where the mother is the infant's holding environment; she meets the needs of the infant and relates to them but also retreats when there is no need for a prompt response (Winnicott, 2009). In the same way this relationship is reconstructed in therapy; Winnicott contended that in psychoanalysis the therapist must provide the patient with holding, meet his real needs, and thus form an environment that helps the psyche develop. Spitz (2021) adds that the literary text too may be a holding environment for its reader. Namely, both the literary text and bibliotherapy are a holding environment.

#### Children's writing as subversion against the adult world

As stated, children's writing reflects their mood, fields of interest, manners of coping, and personal language (Sela and Raz, 2019; Segev-Shoham, 1998; Sunderland, 2017; Suvilehto, 2016). However, it also does something else. In my previous study (Ifrah, 2024), I claimed that children's writing is subversive in essence. I described a course where, similar to feminist theory that indicates women's writing as subverting patriarchal authority (Irigaray, 2004; Cixous, 2006; Cixous and Clement, 2006; Showalter, 2006), children's writing subverts the law of adults. According to Lacan (2015), the child relinquishes the fantasy of uniting with the mother by grasping the existence of the father, who represents regulating and organizing functions of language and of the law (Mitchell and Black, 2006; Miller, 2006).

Lacan offers a new interpretation of the Oedipal complex. He posits that the child who experiences a state of merging with his mother begins to recognize his separateness from her. He continues to yearn for the mother and for union with her, a craving that Lacan calls desire. This is the desire to be everything for the mother (Lacan, 2015). However, he can't be because the father is the one who has the phallus, he is the mother's partner. Hence, the child who lacks the father's phallus is emasculated. Therefore, the child's relinquishing of the dyad and merging with the mother is obtained by the father's presence, who represents the regulating and organizing functions of language and of law [Lacan in Mitchell and Black (2006)]. The child is inducted into the secrets of the language and of the law through the qualities of the language that are regulated by the father and thus recognizes the symbolism of the relations and how they can be realized in practice only through language [Lacan in Mitchell and Black (2006) and Miller (2006)]. Namely, the child's access to language occurs at the oedipal stage by understanding that he lacks the phallus, i.e., the capacity to signify.

I claimed that this exclusion from the linguistic order of the adult world requires the child to be active vs. his representation, which is why children's writing subverts the adult's symbolic order. The law of the father is the law of the adult, whether man or woman, which requires the child to make adjustments to enter the language. Therefore, the child's ability to enter the symbolic order through writing is a significant and active manner of subversion. A writing child subverts the adult's hegemony over formulating, speaking, and investigating the child, and offers other ways and possibilities of formulating subjectivity. Being in language.

For David, writing expressed subversion of the law of the adult, who represents social conventions and norms that do not favorably view an occupation with taboos such as bodily excretions. Writing allowed him to challenge the distinction between that which is a proper topic of discussion and that which is not, and with the support of the literary work, his disruption of orders and norms was legitimized. Both the literary text and creative writing transform the infantile, uncommunicative, and non-normative parts.

In a wider sense, the characters he chose to write, a doctor and a captain, reflect a projection of the practitioner/client relationship. Moreover, the ever-present question—Will I accept him despite the disgust and stink? Will I love him with his many non-perfect parts although he does not toe the line, is not like everyone else, is **confusing, annoying, and hypnotizing?**

## Discussion and conclusion

Processes of writing and reading within bibliotherapy establish a space for exploration and pausation. For David who is in the latency stage, the occupation with age-inappropriate contents is aimed at regressing for the purpose of leaping forward. These contents receive a presence and reference through the literary text *Captain Underpants* which deals humorously with social taboos related to bodily excretions, thus serving as a “holding environment” (Spitz, 2021). In addition, the analytic environment serves as a holding and transforming environment (Bollas, 2018) that allows the child to explore these areas of the psyche and be transformed by them. Our relationship gives David the confidence and the ability to be present relatively safely in primary, non-verbalized, and uncomprehended places. He exits them stronger and with the ability to communicate himself verbally in direct conversation with me.

When Winnicott (2009) wrote about play and creativity, he placed them within the protected area that the potential space gives the individual. There is the external reality that is usually constant, and it is one's contact with the external world. Within this relationship one learns rules and norms and internalizes them, as well as taboos, where engaging in them hampers efficient communication. At the other extreme is the internal reality, which belongs to one privately, the autarchic economy where one's hidden life is conducted. Between the two is the potential space. David addresses the contents that overwhelm him within the protected therapeutic space. Within this space, the literary text and creative writing are the bodyguards of the id's urges, in the manner that the contents and urges that overwhelm the psyche receive aesthetic meaning.

### The implications of the study for educational, therapeutic, and health practices

The implications for the therapeutic field include unique manners of bibliotherapy, i.e., the use of literary works alongside creative writing in the treatment room, focusing on a unique psychological phenomenon common in therapy with children. This is valid for therapy with children in general and for children who are reluctant to work with art materials in particular.

The implications for the educational field involve providing a wide frame of reference for the educational field focusing on regressive stages in children. In this way, the study enriches educators' toolbox for handling this phenomenon. In addition, the study offers a new perspective for psychologists and psychotherapists who work with children.

### Research limitations

The research limitations are related to the study's utilization of several case descriptions merged to form one illustrative case. This was done to maintain ethics in research by adhering to the principle of patients' privacy. In addition, a composite case description focuses on a certain idea, whereas in the current study this is the process of regression in the service of the ego. Nonetheless, possible limitations of this method are that it does not reflect the complete therapeutic process of a single patient. In addition, the data are limited and might thus raise questions concerning the required generalizations from this study.

## Footnotes

^3^In the second part (the second of the 12-book series), Harold and George continue to write the comics story; this time they write *Captain Underpants and the Attack of the Talking Toilets*, where Captain Underpants is Mr. Krupp, the hated school principal. Whenever someone snaps his fingers, the principal unknowingly turns into Captain Underpants.
